# SARS-CoV-2 Infection and the Male Reproductive System: A Brief Review

**DOI:** 10.3390/life13020586

**Published:** 2023-02-20

**Authors:** Vittoria Rago, Anna Perri

**Affiliations:** 1 Department of Pharmacy, Health and Nutritional Sciences, University of Calabria, 87036 Rende, CS, Italy; 2 Department of Experimental and Clinical Medicine, University of Catanzaro “Magna Græcia”, 88100 Catanzaro, CZ, Italy

**Keywords:** SARS-CoV-2, COVID-19, male reproductive system, fertility

## Abstract

Many studies have suggested that SARS-CoV-2, directly or indirectly, can affect the male reproductive system, although the underlined mechanisms have not been completely elucidated yet. The purpose of this review is to provide a summary of the current data concerning the impact of SARS-CoV-2 infection on the male urogenital tract, with a particular emphasis on the testes and male fertility. The main data regarding the morphological alterations in the testes emerged from autoptic studies that revealed interstitial congestion, micro thrombosis, reduction of Sertoli, Leydig, and germinal cells, infiltrated immune cells, and atrophic seminiferous tubules consistent with orchitis. Furthermore, men with severe infection exhibit sperm parameter alterations, together with abnormalities of the hypothalamic–pituitary–testis axis, strongly suggesting that SARS-CoV-2 could increase the risk of male infertility. However, despite the inadequate number of longitudinal studies, spermatogenesis and sex hormone imbalance seem to improve after infection resolution. The yet unresolved question is whether the virus acts in a direct or/and indirect manner, as discordant data related to its presence in the testis and semen have been reported. Regardless of the direct effect, it has been postulated that the cytokine storm and the related local and systemic inflammation could strongly contribute to the onset of testis dysfunction, leading to male infertility. Therefore, multicentric and longitudinal studies involving a large number of patients are needed to understand the real impact of SARS-CoV-2 infection on male reproduction.

## 1. Introduction

**Figure 1:**
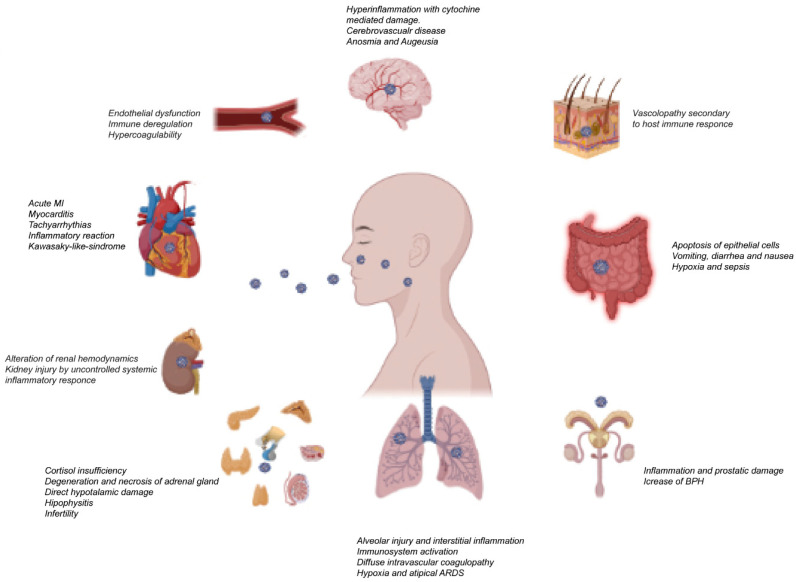
Main effects of SARS-CoV-2 infection in human tissues. Myocardial infarction (*MI*); benign prostatic hyperplasia (*BPH*); acute respiratory distress syndrome (*ARDS*).

The unexpected pandemic outbreak of SARS-CoV-2 has brought unpredictable challenges worldwide, increasing deaths and financial losses. As is already known, to enter into host cells, SARS-CoV-2 uses the Angiotensin 2 Converting Enzyme (ACE2), by which the virus binds to the cell, and transmembrane serine protease 2 (TMPRSS2) cleaves the viral spike protein, allowing the release of viral RNA into the cell [1]. In cells that do not express TMPRSS2 or where the expression is too low, the virus enters through the endosomal pathway, using ACE2 and cathepsins [2]. ACE2 protein is highly expressed in the upper and lower respiratory tract cells and recent in vitro studies demonstrated that a higher expression of the ACE2 gene is associated with a higher risk of SARS-CoV-2 infection [3,4]. Furthermore, many factors, such as environment, living habits, gender, age, BMI, and epigenetic mechanisms, could modulate the cellular expression level of ACE2, increasing or decreasing the susceptibility to COVID-19 [5]. This concept has been further strengthened by the study of Bartolomeo et al., demonstrating that ACE2 overexpression changes the SARS-CoV-2 kinetics in pulmonary cells, increasing the SARS-CoV-2 replication capacity [6]. In addition to being highly expressed in lung cells, ACE2 is also expressed in different tissues, such as the digestive, circulatory, central nervous, urogenital, and reproductive systems, that, therefore, can be directly infected by SARS-CoV-2 [7] (Figure 1). However, the findings that demonstrate the presence of the virus in different organs mainly emerged from autoptic studies, in which the value is limited by many factors, such as sample degradability and contamination, and the absence of positive controls, leading to testes’ misinterpretations [8,9,10,11]. Nonetheless, vast evidence has demonstrated that SARS-CoV-2 infection causes severe pulmonary involvement, concomitantly influencing different organs, leading to the critical situation of multi-organ failure, which might advance to a condition called “cytokine storm” mainly observed in the severe form of COVID-19 [12]. However, the molecular pathology of multi-organ injuries in COVID-19 patients remains unclear. For this reason, the use of infected animal models could allow a more in-depth investigation of the affected tissues. To this end, a recent preliminary study conducted on Macaques has given indications about systemic infection by SARS-CoV-2 [13].

Epidemiological studies have reported that men are more likely to be infected with ASARS-CoV-2, developing more severe symptoms than women [14,15]. The ACE2 levels in males are higher than females [16]. In contrast, TMPRSS2 does not seem to be associated with a significant sex-related differential expression in humans [17]. Sex disparity in susceptibility and outcome of COVID-19 seems to be due to different mechanisms, including sex hormone influence on factors that facilitate virus entry and priming, the immune and inflammatory response, as well as coagulation and thrombosis diathesis. Furthermore, men have a less effective immune response with consequent severe clinical manifestations of the disease [18].

Several authors, albeit reporting conflicting results, highlighted the negative effects of SARS-CoV-2 infection on the male reproductive system, although, to date, the underlying mechanisms have not yet been completely elucidated. Studies using public single-cell RNA sequencing datasets reported a high expression level of ACE2 and TMPRSS2 in the testis including the spermatogonia, peritubular myoid cells, testis somatic cells, and spermatogonial stem cells [19], but similar studies did not find ACE2/TMPRSS2 co-expression in any type of testicular tissue [20], suggesting that testicular cells do not have a high risk of viral entry and infection and that the impaired testicular function could occur through indirect mechanisms, such as high fever and inflammation promoted by the cytokine storm. Furthermore, some studies showed that SARS-CoV-2, similar to other viruses including HIN1, Zika, HIV, hepatitis, and papilloma, could alter testicular function by promoting an oxido-inflammatory response, atrophy of the seminiferous tubules and Sertoli cells, and reduced Leydig cell mass with hypotestosteronemia [21]. In addition, several studies have demonstrated that some viruses can be sexually transmitted because they evade the host immune response via extracellular vehicles (EVs). However, the investigation whether EVs protect SARS-CoV-2 virions from testicular immune surveillance [22] is an issue to be addressed. However, the topic of sexual transmission of SARS-CoV-2 remains an open question, as the available studies are controversial [23]. Nonetheless, the published data on the negative impact of SARS-CoV-2 infection and male infertility are more consistent, although detectable changes in semen analysis, such as decreased sperm count, increased morphological changes, decreased motility, and increased DNA fragmentation, and alteration in the balance of sex hormone have been demonstrated. Further studies are needed to better understand the underlying molecular mechanisms, as well to establish the length of the damage persistence.

The purpose of this review is to provide a brief summary of the current knowledge on the impact of SARS-CoV-2 infection on the male urogenital tract, particularly on the testes, resulting in the reduced testicular function, such as impaired spermatogenesis and reduced testosterone production.

## 2. SARS-CoV-2 Infection and the Prostate

The studies reported in the literature demonstrated that components of the renin-angiotensin system are present in the prostate gland and that the expression of ACE and Ang-II are markedly increased in patients with benign prostatic hyperplasia (BPH) [24,25,26]. Some authors reported that there is a correlation between BPH development and prostatic inflammation promoted by a bacterial or viral infection and that the intra-prostatic inflammatory milieu observed in prostate tissue specimens of patients with BPH is sustained by infiltrated macrophages, i.e., B and T-cell lymphocytes, secreting many pro-inflammatory cytokines [27,28]. It is well known that ACE2 and its product Ang (1–7) exert antiproliferative and anti-inflammatory effects, modulating leukocyte infiltration and cytokine secretion by counterbalancing the Ang-II effects [29]. Therefore, as SARS-CoV-2 induces the suppression of ACE2 to gain entry to the target cells, it can be hypothesized that the virus infection, by promoting the activation of pro-inflammatory pathways, can lead to the development of irritative symptoms of BPH and triggering an inflammatory process in the prostate gland. Starting from this evidence, considering that BPH is more common among elderly men, who also present a higher susceptibility to COVID19, careful urological monitoring of these subjects during infection should be recommended.

Moreover, several studies reported that prostate cancer patients with COVID-19 had higher hospitalization and mortality levels compared with patients with non-prostate genitourinary cancer (bladder and kidney) and other solid cancers, highlighting the importance to implement effective measures for clinical management of these patients during the pandemic [30,31,32]. The unfavorable clinical outcome observed in these patients seems to be strongly influenced by the androgen receptor signaling, a hallmark of prostate cancer, as androgens enhance the expression of the type II protease, TMPRSS2, and suppress the innate and adaptive immune responses [1,33,34]. However, in addition to these molecular factors, other conditions, such as old age, diabetes, hypertension, and behavioral factors, e.g., smoking, can increase the exposure to the COVID-19 risk for prostate cancer patients [34]. Interestingly, the recent study of Raza MT et al. found that TMPRSS2 and the cytokine CXCL10 resulted over and co-expression in prostate cancer and COVID-19, and that they can represent two putative biomarkers responsible for the increased vulnerability and fatality of prostate cancer patients to COVID-19 [35].

## 3. SARS-CoV-2 Infection and the Testis

The interest of the researchers toward the possible influences of COVID-19 on male fertility arises from the evidence that testis express ACE2 and TMPRSS2, including spermatogonia, peritubular myoid cells, testis somatic cells, and spermatogonial stem cells [36]. However, studies based on single-cell RNA sequencing did not find ACE2/TMPRSS2 co-expression in any type of testicular tissue [20], hence, the direct capacity of SARS-CoV-2 to promote direct testicular damage is a still debated issue. Notably, a recent study reported that when TMPRSS2 is not expressed or has low expression, SARS-CoV-2 enters the host cells through the endosomal pathway, using ACE2 and cathepsins [2]. In addition, inflammatory cytokines and oxidative stress SARS-CoV-2-induced can affect Leydig cells, disrupting their capacity to produce testosterone, and germinal cells, hindering spermatogenesis [37]. Finally, Geslot, A et al. described a hypothalamic pathology resulting from SARS-CoV-2-related neuroinflammation, which can alter testicular function, affecting gonadotropins secretion [38].

On this ground, it is reasonable that SARS-CoV-2 could affect testicular physiology and consequently male fertility through different ways.

**Figure 2:**
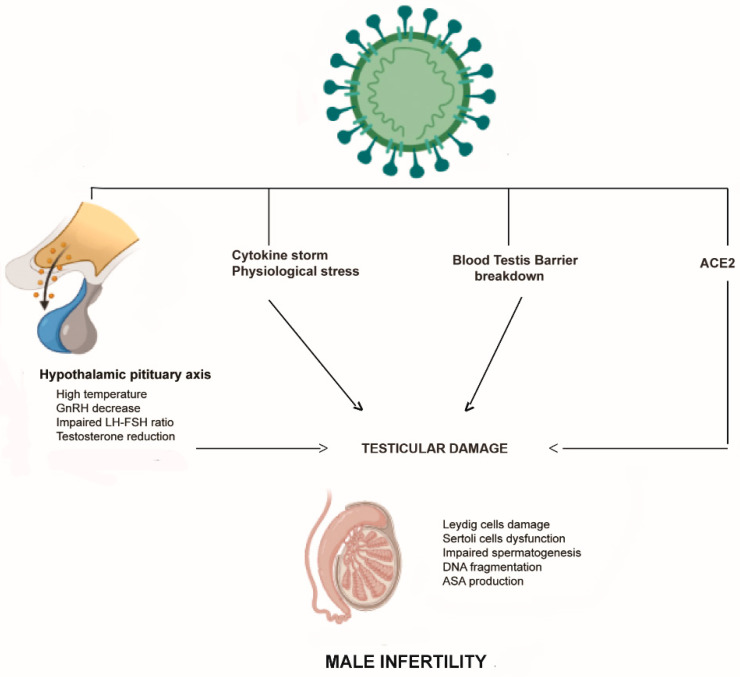
Possible direct and indirect mechanisms of action of SARS-CoV-2 on the testicular function, leading to male infertility. Gonadotropin-releasing hormone (GnRH); luteinizing hormone (LH); follicle-stimulating hormone (FSH); angiotensin 2 converting enzyme (ACE2); anti-sperm-antibodies (ASA).

The data regarding the impact of SARS-CoV-2 infection on testicular function mainly arises from a few autoptic studies performed on testis and epididymis specimens of dead patients with COVID-19. Although the evidence regarding the presence of the virus in the testes is remarkably discordant, unanimous information is available on the macro and microscopic damage to the testes. The study of Ma et al. evaluated the detrimental effects of SARS-CoV-2 infection by examining the molecular features of testes obtained from COVID-19 patients [39]. The authors found numerous apoptotic germ cells concomitant with a significant number of infiltrated immune cells in the interstitial compartments, and they also observed plasma cells and activated B cells, suggesting that SARS-CoV-2 might trigger a secondary autoimmune response, in addition to the primary pathogenesis of viral orchitis [39]. Furthermore, the authors found an elevated expression of ACE2 and TMPRSS2 in the seminiferous tubules of all patients, supporting the hypothesis that SARS-CoV-2 could directly attack testicular cells. Finally, they evaluated the transcriptome changes in all patients’ testes, establishing, by Gene Ontology analysis, the activation of some inflammation-related processes and the downregulation of genes implicated in spermatogenesis [39]. The hypothesis that the elevated testicular immune response could cause impaired sperm parameters and testicular damage, has been further supported by the study of Li et al., who reported edema, interstitial congestion, and red blood cells at the autopsy of testicular and epididymal samples from patients who died from COVID-19 [40]. Moreover, the authors found elevated seminal levels of IL-6 and TNF-α, suggesting an autoimmune origin of orchitis [40]. Extensive germ cell damage has been also described by Yang et al., who concomitantly observed detachment of Sertoli cells from the tubular basement membrane, leukocyte infiltration, and a reduced number of Leydig cells [41]. Similar results have been reported by other autoptic studies showing signs of acute damage, including sloughing of spermatocytes, swelling of Sertoli cells, microthrombosis in the testicular vasculature, increase in apoptotic cells within seminiferous tubules, and increased infiltration of CD3+ T lymphocytes and CD68+ macrophages in testicular interstitium [40,41,42]. Interestingly, the retrospective study of Chen et al., including 142 patients positive for COVID-19 who underwent scrotal ultrasound at diagnosis, revealed that about 22% were found to have increased tunica thickness and increased vascular flow consistent with orchitis, epididymitis, or epididymo-orchitis [43]. Overall, the aforementioned studies strengthened the concept that inflammation plays a crucial role in testis damage observed in patients with COVID-19, and in this regard, an important role has been addressed in innate immune system activation [44]. It is worth considering, in addition to the potential SARS-CoV-2 direct testicular damage, that the cytokine storm triggered by infection could disrupt the blood–testis barrier, as already reported [45,46], playing a critical role in the development of orchitis. The latter not only disrupts the immune balance of the testis microenvironment, but also promotes detrimental effects on the seminiferous epithelium and spermatogonial stem cells, concomitantly increasing the probability of the virus entering the testis (Figure 2).

Overall, the testicular changes observed in patients who died with COVID-19 could negatively affect testicular function, both in terms of testosterone production and spermatogenesis, leading to male infertility. To our knowledge, no autoptic studies have explored whether some of the above described testicular changes are detectable even long after the previous SARS-CoV-2 infection, as already reported for multiple organ systems, additionally highlighting the importance of vaccination to counteract the severity of the damage [47].

### 3.1. SARS-CoV-2 in the Semen and Sperm Parameters in COVID-19 Patients

The presence of SARS-CoV-2 in the semen represents a noteworthy, debated topic in the literature. Two recent systematic reviews and meta-analyses reported that SARS-CoV-2 viral RNA is undetectable in the semen samples of COVID-19 patients with active infections or in those who have recovered; conversely, only two studies documented semen viral mRNA detection in a relevant number of patients [48,49]. With respect to this topic, Corona G et al. speculated that the seminal identification of the virus in the early phase of the infection can be the consequence of the alteration of the blood–testicular barrier, or its excretion in the seminal fluid. In addition, the authors highlighted that the majority of available studies provided limited information regarding the method of semen collection and preparation. It is well known that a correct semen sampling method is crucial, as the detection of virus in the semen could reflect contamination from feces, urine, hands, or respiratory droplets [50]. The sensitivity and specificity of the RT-PCR methods used to detect SARS-CoV-2 in seminal fluid is a very important issue [51]. Paoli D et al. performed a new qualitative determination of the RT-PCR assays in different fractions of seminal fluid from patients with COVID-19, confirming the feasibility of this test for the molecular diagnosis of SARS-CoV-2 in seminal fluid [52]. The potential risk of sexual transmission and semen contamination of SARS-CoV-2 remains an open question, being particularly relevant for the cryopreservation and use of male gametes in Assisted Reproduction Technology (ART) procedures. Currently, although no global guidelines are provided, as well as data on the minimum required interval between COVID-19 recovery and ART, many scientific societies affirmed the importance of continued reproductive care during the COVID-19 pandemic, concomitantly recommending caution to couples planning natural pregnancy or ART [53].

The studies evaluating the effects of COVID-19 on semen parameters compared to healthy controls demonstrated that COVID-19 was associated with a significant reduction of total sperm count, sperm concentration, and total motility, together with a lower seminal volume, whereas no difference in sperm morphology or progressive motility was observed [48]. Conversely, the results of Pazir’s study conducted among 24 subjects before and after SARS-CoV-2 infection demonstrated a significant decrease in total motility and total motile sperm count [54].

Interestingly, Hajizadeh Maleki et al. found higher levels of seminal ACE2 enzymatic activity, oxidative stress, and pro-and anti-inflammatory cytokines. In addition, the authors observed considerable cytopathological alterations, DNA damage, and apoptosis in the sperm cells, amounting to a transient state of male subfertility such as that with oligoasthenoteratozoospermia. The further significant finding emerging from this study is that the above-reported semen alterations persisted over time, reinforcing the need for a more extended period to recover the human male reproductive system’s immune responses after COVID-19 infection [37,55]. Similarly, the study of Li et al., which has been conducted among 23 hospitalized COVID-19 patients, demonstrated that more than one-third have sperm density reduction concomitant with increased levels of leukocytes and inflammatory factors compared to controls [40]. Furthermore, the disruption of the blood–testis barrier involving Sertoli cells caused by the cytokine storm could promote the production of anti-sperm antibodies, whose presence is known to be associated with lower sperm concentration and motility [56]. The recent meta-analysis conducted by Yudhistira Pradnyan Kloping et al. compared the results between patients with different degrees of severity, showing that the patients with more severe infections had worse sperm analysis results, both in concentration between mild and moderate, mild and severe, as well as moderate and severe infections, and mean semen volume between moderate and severe infections [49]. The reasons behind these findings may depend on different and concomitant factors, including the direct local damage caused by the virus and the systemic effect. In particular, the elevated body temperature observed in severe cases, as well as the cytokine storm, promote deleterious effects on sperm count and motility [57,58,59,60]. The study conducted by Donders et al. reported that sperm parameters were mostly severely damaged when assessed during the first month after COVID-19 infection and were less pronounced and almost normal in men tested after more than 1 and 2 months, respectively, suggesting a tendency of COVID-19-related sperm impairment towards reversibility [61]. In this regard, Paoli D et al. performed a seminological evaluation three months after COVID-19 recovery, demonstrating that semen parameters and sperm DNA fragmentation presented no significant long-term impairment, and no sperm autoimmune response was detected. The authors stress the concept that SARS-CoV-2 can temporarily affect spermatogenesis and that when sperm analysis is conducted after a complete spermatogenetic cycle, during approximately 78 days, alterations in seminal parameters are no longer noticeable. Against this background, the authors suggested that it is possible to counsel infertile couples to postpone ART procedures for around three months after recovery from the infection [62].

One limitation of the studies exploring the changes in semen quality in the above-reported patients with COVID-19 is the lack of pre-infection data since the referent point is the comparisons with uninfected controls. Furthermore, an important topic is how long after infection the seminal changes persist. A new scenario seems to be opened by recent studies demonstrating recovery at three months after infection. Therefore, similar further multicentric studies in that regard should be performed in order to confirm that the seminological changes are transient, as well as the general damage of the testes.

In addition, these studies should be designed to better establish whether and to what extent the changes correlate with the severity degree, strengthening the relevance of SARS-CoV-2-related systemic inflammation in the development of the testicular dysfunction.

### 3.2. COVID-19 Vaccination and Semen Parameters

Recently, the possible impact of COVID-19 vaccination on semen parameters in healthy men appears to be a remarkable concern. Gonzalez et al. reported that mRNA vaccines (BNT162b2/Pfizer-BioNTech and mRNA-1273/Moderna) did not promote statistically significant changes in any sperm parameters after vaccination [63]. Similar results have been obtained by Safrai et al., although it is important to note that this study was published as a preprint and has not been peer-reviewed [64]. The recent longitudinal study of Gat et al. aimed to investigate the effect of the COVID-19 BNT162b2 (Pfizer) vaccine on semen parameters among semen donors. The authors demonstrated a selective temporary decline in sperm concentration and total motile count three months post-vaccination, followed by recovery, strengthening the need for further studies on different vaccines and populations, particularly in sub-fertile patients [65]. Finally, the recent narrative review of Pourmasumi et al. reported that no impairment of reproductive function caused by vaccines has been demonstrated [66]. Therefore, starting from the evidence emerging from the available clinical trials, the medical community should reassure the public about the safety of vaccination to prevent the negative effects of COVID-19 also on male fertility.

### 3.3. Hypothalamic–Pituitary–Testes in COVID-19 Patients

A further aspect that should be considered is that SARS-CoV-2 has been shown to cross the blood–brain barrier, and when infecting ACE2-expressing cells, it promotes neuroinflammation in brain regions, including the hypothalamus, leading to the disruption of its physiological functions of temperature regulation and hormone balance [67,68]. Therefore, the increased risk of male infertility observed in patients with COVID-19 could also be addressed by the dysfunction of the hypothalamic–pituitary–testes axis, which causes the abnormal secretion of GnRH, LH, and FSH, affecting testosterone production and spermatogenesis. However, although hypogonadism resulting from inflammation in the testes is increasingly evident [69,70], to date, few studies have investigated the contribution of hypothalamic dysfunction. Some studies report increased circulating levels of gonadotropins in men with COVID-19, mainly in those with more severe disease, speculating that these findings could be the consequence of the transient activation of the gonadotropin-secreting cells due to early inflammatory responses [71,72]. In addition, it should be considered that damaged Leydig cells after SARS-CoV-2 infection can reduce testosterone secretion, which may eventually lead to an increase in gonadotropin levels through pituitary feedback. Researchers have evaluated the androgen circulating levels in COVID-19 patients, sometimes showing different results. In contrast to the analysis proposed by Ma et al., in which no statistical difference in testosterone levels in COVID-19 men compared to non-infected control men have been observed, Rastrelli et al. observed that men with severe COVID-19 infection or dead patients have lower serum testosterone levels than men who have recovered clinically [73]. Similar findings emerge from the retrospective study of Schroeder et al., showing that severe COVID-19 in men is associated with reduced androgen levels [74]. Analogously, Kadihasanoglu et al. discovered that serum LH levels are higher in COVID-19 patients than in controls, while testosterone levels are lower and were negatively correlated with hospitalization time [75]. In agreement with the aforementioned studies, the recent prospective offered by Ertas et al. demonstrates that serum testosterone levels are lower and serum LH levels higher in COVID-19 patients when compared to the control group [76]. Finally, the recent systematic review and meta-analysis of Corona G et al., highlights that low testosterone levels observed in the acute phase of the COVID-19 was associated with an increased risk of being admitted to the intensive care unit or death [48].

## 4. Conclusions

Collectively, the studies reported in the literature have demonstrated that SARS-CoV-2 infection can affect the male reproductive system, although the major focus of studies is mainly focused on the short-term complications and acute phase of the infection rather than on long-term complications. Nevertheless, most of these studies show significant limitations, such as small sample size, enrollment of patients with different degrees of COVID19 severity, and the heterogeneity in the study designs. Overall, these factors did not allow us to provide consistent conclusions yet. Furthermore, to date, the mechanisms by which COVID-19 impairs testicular function are highly debated. First of all, it is unclear whether SARS-CoV-2 can directly infect testes cells, since key players mediating the entry of the virus into the cell, ACE2 and TMPRSS2, do not appear to be co-expressed in any testicular cell. Therefore, it has been postulated that a critical indirect pathogenetic role could be exerted by fever and inflammation related to the cytokine storm observed during severe COVID-19, as suggested by studies showing high levels of seminal pro- and anti-inflammatory cytokines, apoptosis markers, and impaired antioxidant activity. In addition, the pro-inflammation status in the male reproductive tract could trigger a local autoimmune response, contributing to testicular damage. Finally, the virus could reach the semen from the blood, through the blood–testis barrier, whose functional integrity could be affected by systemic inflammation. Therefore, the administration of antioxidants in conjunction with drugs counteracting the effects of the cytokine storm should be recommended to prevent testicular injury and to preserve fertility.

A further topic still debated is how long after infection resolution spermatogenesis is restored; although recent studies showed that the detrimental effects of SARS-CoV-2 infection on semen quality could be temporary. A finding related to these issues is essential mainly for couple candidates for ART and the cryopreservation of male gametes. Therefore, ongoing research on the sexual transmissibility and long-term reproductive health of recovered SARS-CoV-2 patients could lead to revisions of public health policies, and provide prevention guidelines, additionally strengthening the importance of vaccination.
